# Claiming ecological grief: Why are we not mourning (more and more publicly) for ecological destruction?

**DOI:** 10.1007/s13280-023-01962-w

**Published:** 2023-12-07

**Authors:** Marzia Varutti

**Affiliations:** Swiss Center for Affective Sciences (CISA), University of Geneva, Campus Biotech, Chemin des Mines 9, 1202 Geneva, Switzerland

**Keywords:** Ecological grief, Emotions, Environmental justice, Mourning, Ritual

## Abstract

Eco-anxiety, grief and despair are increasing, yet these emotions tend to remain private, rarely expressed in public. Why is it important and necessary to grieve for ecological loss? Why are we not—as individuals and societies—coming together to express and share our grief for ecological destruction? I address these questions from three angles. Firstly, I draw on recent literature on ecological grief and prior work on grief for human lives, to argue for the importance and urgency of grieving publicly for ecological loss. Building on this, I identify perceptual, cognitive, affective, ritual and political obstacles to ecological mourning; these obstacles point at critical intersections between emotions, practices, disciplines, public and private realms, which can turn into fruitful venues for further research, debate and action on ecological grief (and its absence). In closing, I propose a set of ‘ecological skills’ that might help us overcome these obstacles, and lead us to embrace ecological grief and mourning as acts of ethical responsibility and care for the planet.

## Introduction

Despite the increasing pace and scale of ecological destruction, societal response remains largely characterized by indifference. Confronted daily with evidence of irretrievable ecological loss, we are turning our heads away and carrying on with our lives almost as if nothing were. Yet normalcy is an illusion, as our emotional turmoil can no longer be concealed: eco-anxiety, anger, depression and lack of hope in the future are on the rise, especially among young generations (Comtesse et al. 2021; Ojala et al. 2021). Stifling the expression of pain is detrimental not only for the individual but also for society at large. It is problematic if the emotional distress caused by ecological degradation remains private, unnoticed or treated as an individual, rather than a societal issue. Instead, if we express our grief in public settings and share it with others, it becomes visible as a societal legitimate response to a common, planetary problem (Cunsolo and Landman 2017, p. 5; Gillespie 2020, p. 56).

The key premise of this article is that grieving and mourning for ecological loss are necessary. They are necessary because they bring us to acknowledge the loss and reflect upon it, thus countering denial and lack of attention (key culprits of our collective inaction). They are also necessary because they allow us to deal productively with the negative emotions that paralyze us, such as sorrow, anxiety, guilt, anger and despair: we can express and share these emotions with others; this can lead to healing as well as pro-environmental decision-making. Through ritualized, collective practices, mourning transforms these negative emotions into a force of change, a societal and political impetus that moves from the higher ethical grounds of reciprocity, responsibility and care.

The article unpacks the arguments for the necessity and urgency for ecological mourning and sets them against our generalized indifference: why are we not—as individuals and societies—coming together (more and more publicly) to express and share our feelings for the ongoing ecological destruction? What restrains us from acknowledging and sharing with others the emotional distress we clearly do experience in the face of ecological decline?

As it will become evident from the discussion below, others have raised similar questions before; we must continue to engage with these questions: they are more relevant and pressing than ever, as political and societal lack of concern and mobilization lie at the core of our collective failed response to the crisis. In an effort to make some sense of this emotional, societal and political impasse, I develop arguments in three complementary directions.

Firstly, I offer a theoretical discussion of the concept of ecological grief by inscribing it in the context of the recent, field-defining literature across the environmental humanities, environmental psychology and philosophy (e.g. Lertzman 2015; Cunsolo and Landman 2017; Cunsolo and Ellis 2018; Albrecht 2019; Barnett 2019; Jensen 2019; Ojala et al. 2021; Pihkala 2022) and illustrate how these strands of theory connect with prior work on grief for human lives, notably drawing on the seminal work of American philosopher and gender studies scholar Judith Butler (2004, 2009).

I build on this theoretical framework to argue for the importance and urgency of grieving publicly for ecological loss, understood as a practice that is psychologically healthy, socially galvanizing, politically legitimate, environmentally sound, and ethically imperative.

In a second moment, I leverage on the theory to identify a series of obstacles and reasons why we are not grieving more, and more publicly, for ecological losses. I specifically draw on the literature to make a connection between the reasons for not mourning human lives, and the reasons for not mourning nonhuman losses. This leads me to identify a set of perceptual, cognitive, affective, ritual, and political obstacles to public mourning of ecological loss. These obstacles, far from comprehensive, point at critical intersections between emotions, practices, disciplines, public and private realms, which can turn into fruitful venues for further research, debate and action on ecological grief (and its absence). Ecological behavior and decision-making are informed by complex interrelations of affective, cognitive, and socio-cultural dynamics, therefore their study calls for trans- and inter-disciplinary approaches that combine environmental humanities with affective studies, ecopsychology, and environmental philosophy among others. A better understanding of what hinders ecological awareness and action provides a springboard for the development of tools and strategies to act on those obstacles and turn negative emotions such as anger and guilt into something positive for the individual (such as emotional resilience) and the collective (motivation to environmental action). With this in mind, in the last section I offer some perspectives—which I frame as ‘ecological skills’—that might help us remove or overcome obstacles, and embrace ecological grief and mourning as acts of ethical responsibility and care for the planet.

## Ecological grief: theoretical frameworks

The American Psychological Association (APA) produced a report in 2017 entitled *Mental Health and Our Changing Climate* (Clayton et al. 2017) discussing the mental health impacts of the crisis. On the basis of the report, Jensen (2019, p. 132) identifies “three prominent patterns of response to awareness of pressing ecological issues: acceptance leading to debilitating despair, acknowledgment via avoidance, and acceptance leading to active address of core causes, supplemented with emotional and psychological support mechanisms”. Each of these patterns is characterized by complex emotional responses, which may include among others, sadness, connected with a passive acceptance and resignation (Ojala et al. 2021, p. 38); worry and anxiety, future-oriented defense mechanisms that enable us to optimize our decision-making in view of anticipated future threats (Ojala et al. 2021, p. 38); solastalgia, the distress produced by environmental change impacting on people directly connected to their home environment (Albrecht et al. 2007, p. 95); environmental guilt, other-oriented feelings of shame with doing something wrong for the environment (Pihkala 2022; Jensen 2019). These are just a few examples which illustrate the complexity of nuances in our emotional responses to the ecological catastrophe.^1^ This complexity becomes even more patent in the case of ecological grief.

Ecological grief has been defined as “the grief felt in relation to experienced or anticipated ecological losses, including the loss of species, ecosystems and meaningful landscapes due to acute or chronic environmental change” (Cunsolo and Ellis 2018, p. 275).^2^ Ecological grief is typically due to a combination of tangible loss (such as the disappearance of a species or ecosystem), intangible loss (such as the loss of identity ensuing from the degradation of an affectingly charged landscape, what philosopher Glenn Albrecht defined solastalgia, 2005), and anticipatory grief for future loss (Cunsolo and Ellis 2018; Comtesse et al. 2021). Ecological grief is therefore characterized by the convergence of different emotional states, both past and future-oriented, based on both reality and projection, and engendering a deep sense of diminishment and uncertainty. Notably, ecological grief is embodied (it is felt in the body) and connects with practice: one can *perform* grief as part of mourning rituals.

It is useful to bear in mind a terminological distinction between ecological grief and mourning as the obstacles discussed below, while pertaining both the individual experience of grief and its public manifestation as mourning practices, may affect these in slightly different ways. Grief and mourning are often used as synonymous, in fact they largely overlap and feed into each other: grief motivates mourning, and mourning enables the expression of grief. However, a closer look reveals different nuances in terms of temporality and sociality. Grief tends to denote the private, personal dimensions of pain, experienced and expressed mainly at individual level or within a small group of family members and closest friends. Mourning conversely denotes the subsequent and more social phase of sharing the loss with others and the community at large; this entails the longer process of coming to terms with the loss and undergoing a transformation through grief, as individuals and society members. As Barnett (2021, p. 14) concisely put it “our mourning is a way of caring and enacting our concern.” In the same vein, Cunsolo and Ellis (2018, p. 275) explain that “grief is the internal physiological and emotional responses to loss, and mourning is the period of mental, emotional and personal transition as people learn to live again in the context of loss”. Of particular interest is the point of connection between individual, personal grief and collective, ritualized mourning, which Joshua Barnett (2022, p. 5, drawing on Ann Cvetkovich) has called ‘public feelings’, that is “feelings which emerge out of and are sustained by certain forms of social, political, economic and rhetorical life”. The interest of the notion of ‘public feelings’ lies precisely in its overarching quality, its ability to show the connections between personal emotions and public life in ways that highlight how these can mutually influence and shape each other. The discussion of the obstacles to grieving and mourning below, is essentially a discussion of the obstacles in giving expression to public feelings.

What is at stake, when this happens? The pain and sadness that arise when witnessing ecological destruction stem from a sense of connection with the nonhuman world; when this connection is cut or severely compromised, we register it as a sense of loss. Grief is a measure of the connection to what is being lost, a physiological response to the severing of a vital bond. And conversely, societal resistance to (or lack of interest for) grieving and mourning nonhuman loss is revelatory of a broken relationship with the planet. In the words of theologian Douglas Burton-Christie (2011, pp. 30, 39), our inability to mourn manifests “an unraveling of the ties of kinship that bind us to the lives of other beings. (…) a telling, damning comment on our impaired moral condition.” He is echoed by religious studies scholar Lisa Sideris (2020, p. 2) who cautions that “an unwillingness or inability to mourn for the broader spectrum of life bespeaks a worrisome evasion of responsibility for environmental harms.” Public enactments of ecological grief (or lack thereof) can then be seen as prisms for social analysis: they say much about those who do (or do not) engage in them, and who and what we do (or do not) mourn for. As Judith Butler (2004, p. 46) poignantly put it, “I am as much constituted by those I do grieve for as by those whose death I disavow”. It follows that ecological mourning casts light on collective emotions, values, cultural belonging, politics and ethics, and through that, our collective, problematic non-response to ecological demise. It is not surprising that ecological grief is a topic of scholarly interest at the intersection of a range of disciplines—ecopsychology, philosophy, affective sciences and environmental humanities at large.

Ecological mourning ceremonies respond to an ethical call to mourn all lives, human and especially nonhuman (Stanescu 2012); by extending our grief and mourning to nonhumans we acknowledge our own responsibility and implication in the very causes of the loss (Spargo 2004), but we are also making a political statement of dissent, resistance and activism and we are contributing to lay the foundations of a different world, informed by a new ethics. In a sense, as Spargo (2004) has powerfully illustrated, mourning publicly is an act of rebellion, a disruption, a temporary rejection of ‘consolation’, a refusal to restore the status quo and silence consciousness. By keeping the wound open, grieving and mourning keep us alert and responsive to the ethical imperative of not severing the relationship with the nonhuman Other. The Other will be truly lost only when we cut our ties, when the loss is no longer registered and has ceased to inform our present and the way we construct our future.

The work of Butler (2004, 2009), Barnett, Stanescu, Spargo, and Cunsolo among many others, reveals the productive and transformative dimensions of mourning: far from an act of closure or erasure, mourning ‘makes anew’ the object of loss through remembrance and re-evocation. In the process it remakes ourselves as well, as we undergo a deep transformation from which we emerge ethically changed and politically active. As Jensen (2019, p. 126) noted, “when painful, disorienting feelings of loss experienced by an individual (grief) are worked through in ways that promote reengagement with the world post-loss (mourning), they can become a potent catalyst for activism and expressions of ecological care.”

Devoting (scholarly) attention to, and engaging in ecological mourning is also an ethical call to acknowledge the pain experienced by the many people around the world who are mobilizing around these mourning practices. Cunsolo and Landman (2017, p. 7) effectively formulated this as a call to “allow our theoretical work to catch up with the lived experiences of environmentally based grief and mourning. The grief and mourning that people around the world are already experiencing.”

The full significance and long term implications of ecological grief and mourning are just beginning to be investigated. Writing about eco-anxiety, worry and grief, psychologist Maria Ojala and colleagues (Ojala et al. 2021, p. 35) noted that “there is not much literature examining and summarizing the ways in which these emotions are expressed, to what processes they are related”. Specifically, public manifestations of ecological grief have until now remained largely unexplored (Sideris 2020). Yet, around the world, people are gathering in collective rituals to mourn ecological loss, as in the case of the memorials and funerals for melting glaciers held in Iceland (Johnson 2019), Switzerland (Saldivia 2019) and Mexico (Brito 2021) among others. There are indications that ecological mourning might positively impact on individual psychological well-being and emotional resilience, as well as societal cohesion and pro-environmental decision-making (Cunsolo and Landman 2017; Barnett 2021). Ecological grief and mourning counter denial and provide frameworks to express emotions such as pain or anger, and more generally, slow down to cast perhaps a critical eye on our life choices and their environmental impact. We know that taking part in funerary ceremonies helps process the loss and provides comfort through the shared pain (Selman and Burrell 2020); the same can be said of mourning ceremonies for ecological loss. As mentioned, grief is individual—it is perhaps the most individually-felt and intensely personal of all emotions—but it can be soothed by a shared mourning experience. Research in psychology (Zech and Rimé 2005) suggests that expressing negative emotions may promote emotional recovery. Mourning ceremonies offer opportunities to gather with like-minded people to bereave and remember. There is power in a community gathering of this kind: the power to claim the right to gather and mourn for the planet in the very first instance; the power of a shared intention; and the power of an affective shared practice to generate healing for its participants.

Ecological mourning also raises questions of power in another sense, as it casts light on the unequal distribution of impacts and responsibility for ecological disruptions. I turn to this issue in the next section.

## Emotions and environmental injustice

The ecological crisis is producing new forms of inequality and injustice (e.g. Newton 2009; Nixon 2011). As environmental calamities multiply around the world, it is becoming evident that the most affected are the most deprived and fragile: poor countries, lowest social classes, indigenous peoples, minorities and other marginalized groups and individuals. The damages are both tangible and intangible. In addition to the destruction of home and homeland, there are also the less visible, deeply cutting psychological and emotional costs of the disasters.

Increasing awareness of the far-reaching economic and non-economic effects of ecological degradation is fostering debate on the foundational notion of loss. Some scientists (Roe et al. 2023) are suggesting the creation of an international fund to specifically compensate poorer countries for biodiversity loss, following the principles of ‘loss and damage’ and ‘consumer pays’ already evoked in international agreements on climate change. The proposition to extend these principles to biodiversity loss opens interesting debates around the quantification of the loss and the mechanisms of compensation, and thus contributes to strengthen the calls for international negotiation addressing environmental justice. Here again, public mourning can have an impact. As Cunsolo and Ellis (2018, p. 279) note, the “total ‘cost’ of climate change impacts tend to be undervalued, particularly for peoples whose sense of wealth is derived from the intangible—rather than the economic—value of nature. Making explicit the grief experienced as a result of ecological losses may serve to address this inequity and lead to the development of mechanisms that more fully compensate affected people for endured climate related losses.”

The stronger the connections with the land, the stronger the emotional responses to its destruction. Indigenous peoples offer a case in point. In Indigenous lands, histories of colonialism, depredation and discrimination are perpetuated and amplified by the continuous erosion of the natural environments from which the livelihoods and identity of so many indigenous peoples directly depend. Immediate, measurable economic losses for Indigenous communities are compounded by even more damaging ‘invisible losses’: unrecognized damage to Indigenous identities, culture, physical and psychological health and sovereignty (Turner et al. 2008; see also Cunsolo 2017; Cunsolo and Ellis 2018; Middleton et al. 2020; Vecchio et al. 2022). It has been observed that “Indigenous Peoples may experience a distinctive type of ecological grief, one that is entrenched in longer, more complex histories of environmental, cultural, and political loss” (Barnett 2022, p. 9). Likewise, other communities and groups in daily direct contact with the land, such as farmers and environment scientists and activists, by virtue of their attentiveness to changes in ecosystems, are also particularly emotionally affected by the dramatic degradation they witness (Berry et al. 2011; Head and Harada 2017; Barnett 2022).

The unequal distribution of the impacts of ecological collapse calls into question the use of the pronoun ‘we’ when referring to ecological matters. When I use ‘we’ in this article I have two main orders of reference in mind: ‘we’ as humanity, but also and most especially, ‘we’ as consumers in the Global North (Europe and North America). On a most immediate and general level, I intend ‘we’ to refer to the human species (*Homo sapiens*), to underline our collective, global responsibility for life conditions on the planet (in line with Head 2015, among others). Referring to ‘we’ as humanity also acknowledges that loss is a universal experience; I subscribe to Judith Butler’s argument that “it is possible to appeal to a “we,” for all of us have some notion of what it is to have lost somebody. Loss has made a tenuous “we” of us all.” The ‘we’ might be the center of Butler’s concern, but I sense that it’s the word ‘tenuous’ that calls for more critical attention in this sentence. Loss might be a universal experience, yet it is not a strong bond since, as mentioned, not all losses are equal, nor are individuals and communities around the world equally affected. Therefore the ‘we’ of this article more often refers to consumer societies in the Global North—the main contributors to the ecological crisis and the main beneficiaries of the colonial and capitalist systems that fueled it. Yet here again, some groups (wealthy, mostly white, male, urban consumers) are more largely responsible for fossil fuel lifestyles than others. Ecological issues intersect with gender, race, class and social-economic issues, giving rise to new forms of social inequality; while remaining problematically blurred, the pronoun ‘we’ is to be read here as subsuming the complexity of this fractal and unequal responsibility.

## Obstacles to grieving and mourning

This discussion of obstacles to ecological grieving and mourning is theoretically framed by the literature on grief and mourning for human lives (such as the work of Butler 2004, 2009) set here into dialogue with the literature on grief and mourning for ecological loss (e.g. Barnett 2019, 2020, 2021, 2022; Cunsolo and Ellis 2018; Cunsolo and Landman 2017).

In order to mourn publicly for ecological loss, we need to be able and willing to: *perceive* and acknowledge the loss; pause to make sense of it, *understand* its significance and implications; allow ourselves to *feel* it, and *share* those feelings with others as part of ritualized mourning practices. In what follows, I develop a series of perspectives that can contribute to clarify the obstacles hindering each of these steps.

### Perceptual thresholds: ecological degradation as ‘slow violence’

Media coverage of ecological disasters opens sudden, dramatic windows of public awareness about ecological destruction. Sadly, in between attention-catalyst events, public attention tends to fizzle out. The problem is mainly one of lack of visibility in the medium-long term: ecological degradation constitutes a form of ‘slow violence’ (Nixon 2011; Bruns 2021) whereby the damage is continuous, gradual and cumulative, and therefore goes easily unnoticed among a large majority of the population. While we might be aware of losing something—biodiversity; air, soil and water quality; time to change direction—we hardly notice it: the loss is a silent hemorrhage, a quiet fading away of animals and plants that never managed to pierce through our attention. This is fundamentally a perceptual barrier, as our capacity for grieving and mourning is hindered by the impossibility to direct our feelings towards a specific aim (Butler 2009; Barnett 2019).

In part, this barrier can be countered by individuation (singling out the individual from the group). For instance naming an endangered animal individual enables us to relate to it and facilitates emotional engagement (Barnett 2019); we might remember the public commotion fuelled by frenzy media attention around Knut, the captive polar bear held at the Berlin zoo who passed away in 2011 (Manzo 2010; O’Neill 2022). More broadly, the power of individual biographies—whether of an animal, a plant or a mountain—is evident in the literature on extinction, where our emotional responses (or lack of) depend on our ability to establish a relationship with who and what is being lost (see for instance Bonnell and Kheraj 2022). Because naming and individuation can be powerful catalysts of public attention and affection, they enable ‘selective mourning’, that is the selection of who and what we decide can and should be mourned. Anthropologist Sebastian Braun (2017, p. 81) noted that “one of the ways we can adjust to a world so full of loss that grieving becomes impossible is that we can limit who is a relative and what is grievable”. Selective mourning is a coping mechanism, a way to manage the unimaginable vastness of the loss.

This points back to the fact that tragically, most biodiversity loss is faceless and hard to detect. Ongoing important work of public sensitization is carried out by organizations and artists aiming to visualize and substantiate the loss (an example is the initiative ‘Remembrance Day for Lost Species’,^3^ also discussed in Massol de Rebetz 2020). The task is further complicated by the fact that in some instances there is simply little left behind to signify and materialize the past presence. For instance, *Cora Timucua* is a kind of lichen originally found only in Florida, most likely become extinct. All we have as evidence of its past presence are a few specimen preserved in natural history museum collections,^4^ virtually nothing else testifies to the existence on earth of the *Cora Timucua* lichen. For a specimen collected, there are million others lost without trace. Ecological loss can also be masked by the ongoing habitat deterioration (such as soil erosion, oceans acidification, temperature rise) leading to the replacement of one species by another, and thus making it harder to notice the disappearance of the previous species. The paradox of ecological mourning is that it calls us to mourn for something we may have never really known or experienced, or even become aware of having lost. Then grieving and mourning become acts of faith, matters of principle and ethics.

### Cognitive obstacles: the incommensurability of ecological loss

Beyond mourning for the individual ecological loss (the animal, the plant, the place we know) lies the call to mourn for the loss of whole species and ecosystems and, more importantly, for the loss of the *possibility* of establishing a *relationship* with them (Braun 2017). The lack of full knowledge about the earth’s biodiversity and the exquisite complexity of its ecosystems is one of the factors preventing us from appreciating their value, and therefore feeling and mourning their loss. In the words of Burton-Christie (2011, p. 39) “we fail to grieve because we have no knowledge or feeling for these beings”; or as ecologist and philosopher Aldo Leopold (1949, p. 48) famously put it, “we grieve only for what we know”.

When an animal or a plant become extinct or when an ecosystem is radically compromised, what are we losing exactly? The most honest answer is ‘we don’t quite know’, we are confronted with the actual *impossibility* to fully grasp—to assess, imagine and feel—the extension of the loss. Scientists might appear advantageously positioned to evaluate and explain the degree and consequences of ecological loss, yet paradoxically they are the first to warn us about the limits of current scientific knowledge and the impossibility to assess the entity and the implications of the loss. For instance, as the planet’s glaciers are melting, scientists lament the disappearance of high-altitude ecosystems whose internal dynamics and surprisingly rich flora and fauna are only just beginning to be investigated (Gaudio and Gobbi 2022). Awareness of the current fragmentary scientific knowledge about complex ecosystems such as glaciers, makes only more acute scientists’ feelings of loss—a loss that concerns not only previously existing knowledge, but also potential future knowledge. The loss of biodiversity has been compared to the loss of libraries, by virtue of the amount of knowledge lost and the loss of future generations (a thought-provoking discussion of the limits of this metaphor can be found in Sayre 2017). This shows that it is hard but not impossible to feel for the loss of something even though, or precisely because, we still largely ignore it.

This points at a fundamental cognitive obstacle: the sheer vastness of ecological loss makes it hard to apprehend. The quantifiable, knowable loss to this day is daunting, but the real scale of the damage is much larger and difficult to evaluate even for scientists. It is arduous to grasp the full significance of an “average 69% decline in the relative abundance of monitored wildlife populations around the world between 1970 and 2018” (WWF 2022, p. 4). Confronted with the unthinkable extension and depth of the loss, we tend to instinctively retract from the thought as we would from the edges of a vertiginous chasm.

Ecological loss is incommensurable also because it affects, often in unpredictable ways, all aspects of human life. For instance, as the permafrost in Arctic and subarctic regions is slowly thawing, tundra ecosystems and geo-morphology are changing; this landscape metamorphosis is impacting deeply on local knowledge, language and cultural practices (Barry et al. 2013; Pratt and Heyes 2022). In the same vein, in the UK the loss of biodiversity is being reflected in an ongoing impoverishment of the English language as name species and connected terminology fall in disuse and are forgotten. *The Lost Words* (Macfarlane and Morris 2018) was a ground-breaking book project combining scientific dissemination with literary flair and evocative art; the book succeeded in shining a light on the plummeting numbers of well-known UK flora and fauna, and in making a tangible contribution towards bringing these species and their names back into the public imagination, levering specifically on children’s wonder and curiosity. Macfarlane’s statement “we do not care for what we do not know, and on the whole we do not know what we cannot name” (quoted in Flood 2015) clarifies the link between loss of knowledge, loss of language, and collective loss of interest. This has far-reaching consequences: the losses we are failing to see, to feel and to mourn are also the losses of our children and their children, whose lives are diminished by our indifference. In addition, we refuse to take responsibility and acknowledge that a damaged planet is our legacy to them. In this sense, ecological loss is a radical loss of episteme, the deletion of the very possibility of *ever* knowing and experiencing, a severe restriction to everything that we and future generations might have been and become. Contemplating these thoughts not only frustrates our cognitive abilities but also causes deep emotional discomfort; no wonder we are inclined to avoid them.

### Barriers of feeling: the volatility of emotional connections

Deep emotions connect us to the planet and its nonhuman inhabitants; to find evidence of this we don’t need to look further than the emotional bond to our pets or the feelings of awe we experience when hiking and taking in the view from the top. However, often these emotional connections are far from unconditional. Affect for nonhuman beings and landscapes tends to be selective (reserved for specific individuals and places) and/or ephemeral (the awe we experience in situ, on the mountain top, fades away as we return to the familiar topographies of daily life). While we are capable of broad emotional connections with nonhumans in the absence of direct personal engagement with them (for example contributions to wildlife protection initiatives appear to be clearly motivated by emotional responses, Castillo-Huitrón et al. 2020), it is often the case that those emotional connections are not completely spontaneous. In some instances they have to be instilled and nurtured, as in the case of biophilia, “the innate tendency to focus upon life and lifelike forms, and in some instances to affiliate with them emotionally” (Wilson, 2002, p. 134) which can be cultivated among children (Kahn 1997; Kahn and Kellert 2002; White and Stoecklin 2008). In other instances, emotional connections are explicitly stimulated, for instance through glossy wildlife photo calendars, hyperrealist cinematic renditions of climate disasters (Weik von Mossner 2014) or evocative nature writing and poetry (Varutti 2023a). Even more tenuous are emotional connections with entire ecosystems, all the more if perceived as distant from our living environment or hostile (such as high altitude mountains or deserts). It follows that not all ecological losses engender grief: we may respond to some (the loss of a pet for instance) and be impervious to others (the mosquito that bothers us). As Barnett (2022, p. 8) points out “just because we *are* related to such others does not mean that we *feel* connected to them”.

There is nevertheless scope for learning to emote and feel for the planet. There is an element of adaptation in this, as in many ways anthropocenic emotions (the emotions we experience in relation to the ecological crisis, Albrecht 2019; Varutti 2023b) are ‘new’ emotions: as environmental philosopher Glen Albrecht (2017, p. 296) noted “the awareness of human culpability at a global scale is (…) a relatively new experience in the history of human mourning”. At present, our emotional bonds to the planet appear to be tinged of egoism and anthropocentrism: we love the planet because it is … (fill in the adjective of choice: beautiful, unique, ideal…) *for us* human beings. We seem not to have fully internalized our deep interdependence with all the planet’s ecosystems. This crucial awareness cannot be fully attained without acknowledging the ongoing loss, and feeling the pain.

Emotional connection is further hindered by the fact that emotions such as sadness, anger, despair or guilt are highly ambivalent: they may trigger us into action or freeze us in a condition called ‘environmental melancholia’ “in which even those who care deeply about the well-being of ecosystems and future generations are paralyzed to translate such concern into action” (Lertzman 2015, p. 4). Grief is likewise ambivalent. Counter-intuitively, grief is considered a ‘useful’ emotion as it helps us to prepare for other losses. As Parkes and Prigerson (2010, p. 6) note, “the pain of grief is just as much a part of life as the joy of love; it is, perhaps, the price we pay for love, the cost of commitment. To ignore this fact, or to pretend it is not so, is to put on emotional blinkers, which leave us unprepared for the losses that will inevitably occur in our lives and unprepared to help others to cope with the losses in theirs” —a sober reminder of the future-oriented value of ecological mourning. The point is that the diverging valence (the 'positive' or 'negative' character attached to an emotion, Colombetti 2005) of the emotions we experience may pull us at the same time in different directions, ultimately undercutting motivation and action. In our lame emotional responses there might also be an echo of what in the healthcare and humanitarian fields is known as ‘compassion fatigue’ (Ledoux 2015), whereby the over-exposure to the stressful situation (the constant, pressing need to provide care) leads to detachment and indifference. Might it be that we have become so accustomed to accounts of ecological degradation, so drained by their affective intensity, that we no longer register them, nor respond to them emotionally?

Underlying these considerations lies the fact that emoting exposes our vulnerability. Grieving is often perceived as a transitory, as-short-as-possible phase before some kind of resolution: the restoration of a form of ‘order’, the regaining of a real or imagined equilibrium, or the onset of a new phase in our lives. As Butler (2004, p. 21) argued, dwelling in grief amounts to dwelling in a state of vulnerability and uncertainty: “Perhaps mourning has to do with agreeing to undergo a transformation (…) the full result of which one cannot know in advance”. Grief exposes the extent to which we are emotionally related to others, it debunks notions of independence, autonomy and control; full acceptance of this is emotionally demanding and requires considerable emotional maturity. Yet embracing vulnerability might actually transform it into awareness, an asset and a strength. As Butler (2009, p. 43) noted “when a vulnerability is recognized, that recognition has the power to change the meaning and structure of the vulnerability itself.” Accepting vulnerability means developing a deep appreciation for the precariousness of life—its fragile, ephemeral character, whether it is human (Butler 2004) or not (Barnett 2018).

### Paucity of mourning rituals for ecological loss

Societal and cultural contexts deeply affect the public expression of emotions (Ahmed 2013), and by extension, public mourning. Anthropological studies show how virtually all cultures around the world developed rituals to mark the passing of a community member. Vigils, wakes, funerals, obituaries and other similar practices help us make sense of, and commemorate human deaths. Nevertheless, cultural and societal norms in the Global North tend to shun exposure to death and death-related material, and minimize public displays of grief, considered something to be dealt with privately.

Even more problematically, when it comes to nonhuman losses, we (in the Global North) appear to be poorly equipped. Canadian environmental humanities scholar Catriona Mortimer-Sandilands (2010, p. 333) pointedly asks “how does one mourn in the midst of a culture that finds it almost impossible to recognize the value of what has been lost?” This raises another crucial, largely overlooked question: if we are to grieve and mourn ecological losses, how do we do it? There are no established practices. Lamenting the lack of public rituals of ecological mourning, Mortimer-Sandilands (2010, p. 339) notes that “there is no language to express that loss, no collection of shared symbols or rituals to acknowledge the significance of that loss, and certainly no systemic recognition that loss might be (literally) earth-shattering for many people”. Mortimer-Sandilands captures the paradox of being submerged by information about environmental degradation, yet continuing to dwell in ‘an emotional void’ as “there is lots of evidence of environmental loss, but few places in which to experience it *as* loss” (Mortimer-Sandilands 2010, p. 338). As Nancy Menning (2017, p. 39) soberly puts it “the paucity of our rituals and practices to mourn ecological losses is troubling” (in this sense also Barnett 2020; 2022). It is also detrimental since, as Cunsolo and Landman note (2017, p. 17, drawing on Menning) “rituals ease grief, resolve guilt, and can prepare us for the work of environmental activism (…) [mourning] can also direct us to creating a better future.” The lack of established practices to mourn ecological losses contributes to explain why we (Western societies) don’t engage in these rituals. Yet again, this mirrors a questionable ethics revolving around hierarchies of beings and hierarchies of losses, whereby nonhuman losses are simply deemed not worthy of remembrance and celebration.

Yet, mourning practices are gradually emerging around the world to celebrate ecological losses, from tangible commemorative cultural heritage such as the Passenger Pigeon Monument in Wisconsin, to performative ritual practices such funerals for melting glaciers (Johnson 2019). Inspiration and insights on how we might create new rituals for mourning nonhuman losses may also be gleaned from non-Western societies (consider for instance the Lakota communities from the Great Plains who have developed buffalo mourning rituals, Braun 2017), and from artistic practice, as shown by the participatory project ‘Mesosanctuary Mural’ held in December 2022 in Mexico City, which was a collective exploration of ecological grief.^5^ These creative, emotional practices substantiate a new ethics of care and environmental activism; they are central to what Joshua Barnett (2022, p. 146) calls “the difficult yet necessary work of earthly coexistence”.

Below I discuss how the impulse behind these public rituals can be significantly affected by the socio-political context.

### Mourning politics and the non-grievability of ecological loss

Beyond the paucity of rituals, mourning for the environment is further hindered by the political framing of ecological issues as something that does not directly pertain to the individual (considered too small to make any difference) and should rather be left to the marketplace (where the right to pollute is effectively transformed into a commodity) and/or to politics (ecological issues tend to feature prominently in electoral propaganda and political skirmishing, yet seem to routinely fade away after the election).

Political discourse on ecological topics appears to be myopically folded on itself, as political actors are preoccupied more with the reproduction of their own power through the maintenance of established social norms and values, than with facing the fact that the ecological catastrophe is putting all those norms into question. Blühdorn (2015, p. 159) incisively notes “what eco-political discourse is, ultimately, all about, are limits of social acceptability, that is, concerns about violations of established social norms that are deemed unacceptable”. Little wonder then if, as Cunsolo (2017, p. 175) argues, we are *expected not to mourn* for climate change, which has been presented as a non-grievable, non-mournable and a de facto invisible item in the public discourse. To ask whose lives are grievable amounts to ask what is the value we give them. Butler (2009) proposes that if a life is not being grieved, it is because well before its loss, it is already considered as non-valuable, it is already existing outside our matrix of ‘valuable life’, and therefore will not be grieved. Seen in this light, grieving and mourning once again reflect our ethical principles. On the one hand, selective mourning or conspicuous mourning for one loss may indirectly indicate the lesser importance of other losses, thus revealing ‘hierarchies of losses’. On the other, not mourning at all for ecological losses is an active act of derealization of the Other (Butler 2009): by not mourning the nonhuman we negate a relationship with it.

In order to engage with the invisibility of ecological grief, some scholars (Cunsolo and Ellis 2018, p. 275; Barnett 2022, p. 12) have evoked the concept of ‘disenfranchised grief’ (Doka 1989, 2008, p. 223) which refers to “grief that results when a person experiences a significant loss and the resultant grief is not openly acknowledged, socially validated, or publicly mourned. In short, although the individual is experiencing a grief reaction, there is no social recognition that the person has a right to grieve or a claim for social sympathy or support.” Disenfranchised grief echoes Butler’s notion of ‘disavowed mourning’ (2009, p. xiv); both concepts point at what lies beyond and around the object of mourning: a socio-political pact of silence, a tacit censorship that leaves the individual isolated and disempowered in dealing with emotional distress. Current mainstream political and economic approaches to ecological issues fail to fully acknowledge and honor the basic fact that the planet does indeed pertain each and everyone of us, as the ultimate *res-publica.*

In contrast, some voices caution against an excessive focus on developing individual emotional resilience as the most effective way to deal with ecological grief, as this places the responsibility for well-being solely on the individual rather than accepting that this is a societal and political concern. Psychologist Weronika Kałwak and ethicist Vanessa Weihgold (Kałwak and Weihgold 2022) point out that the emphasis on emotional resilience entails a “individualization and responsibilization of individuals against the global and systemic threat of the climate crisis. Discussion on resilience-based interventions in social work calls for bringing back the central place to social issues in professional understanding of mental health (especially in underprivileged populations that are susceptible to climate change impacts in the first place)”. Jensen (2019, p. 136) reinforces this point: “For new modes of ecological mourning to flourish, we must engage environmental guilt’s *collective* dimensions. If feelings of culpability remain coded solely as individual guilt rather than collective guilt, the grief we feel over ecological loss is even more likely to remain individualized” (italics in the original).

Seen in this light, public, collective mourning counters the ‘privatization’ of ecological emotions, that is, it counters the confinement of the emotional burden to the individual and its framing as an individual mental health issue. Instead, ecological public mourning re-places these emotional responses firmly in the public arena, leveraging shared emotions to assert ecology as a key societal and political concern and demanding political accountability.

Mourning can be a powerful tool to shift from despair to action, including civic, political action. Mourning ecological loss amounts an act of activism, a public commitment: it expresses one’s willingness to acknowledge and take responsibility for the loss. As Cunsolo (2017, pp. 172–3) notes, “grief and mourning have the unique potential to expand and transform the discursive spaces around climate change to include not only the lives of people who are grieving because of the changes, but also to value what is being altered, degraded, and harmed as something mournable”. Collective public mourning can then “compel the creation of new understandings of ethico-political strategies for mourning beyond the human based on how nonhuman bodies are created, valued, understood, populated, realized and derealized” (Cunsolo and Landman 2017, pp. 16–17). Deciding what is and is not ‘mournable’ are not just conceptual issues, they are political acts, actionable options that can be implemented and made visible in the public realm through morning enactments, creating precedents and models for further civic and political engagement. Ecological mourning ceremonies not only attest to the grievability of the nonhuman, but also actively constitute it.

The obstacles discussed above—obstacles in perceiving, conceptualizing, feeling and performing our way through ecological loss—are not insurmountable. Their causes can be addressed by a fundamental shift in the way we perceive ourselves as human in relation to the planet. I suggest we can foster this shift through the conscious deployment of a set of skills and abilities that I discuss below.

## Developing ecological skills

What might help soften and ease the obstacles and resistances discussed above? An obvious first step is becoming aware of them. Beyond that, I suggest we need to purposefully cultivate a set of skills, which I call ‘ecological skills’. These are skills that we mostly already have, what makes them ‘ecological’ is that they are newly infused with ecological awareness and directed towards ecological action. They point at a reformed ontology and axiology of being human based on an equal relation with the nonhuman. Ecological skills can be thought of as meta-skills as they underlie and frame other, more concrete and applied sets of skills, tools and approaches which are being proposed to navigate the crisis—such as for instance learning to forge new stories to replace narratives of doom, or learning how to collaborate, more and better, with others (see Ray 2020). The list of skills presented here—briefly considering intimacy, mental flexibility, and creativity—is a point of departure, an invitation for others to join in and identify more ecological skills.

### Intimacy

I approach intimacy as a skill defined by the capacity and the willingness to create the conditions for proximity and connection. In this sense, intimacy encompasses a range of other skills and practices such as attentiveness, patience, perseverance, curiosity, selfishness and trust, among others. If we can narrow down our focus and pay attention, look closely, attend to the unfolding of an event with all our senses, there is an opportunity to expand our awareness of the macro- and micro-scale dynamics of the world around us, establish a connection, and possibly *feel* something. Developing the ability to engage in proximity and intimacy is an antidote to perceptual and affective barriers such as distraction, disconnection, and indifference.

### Mental flexibility

As we are (too) slowly realizing, ending a fossil fuel-based society requires massive, unforeseeable transformations of our lifestyle. Some scholars (Gibson et al. 2015; Head 2015) point out that approaches focused on gradual and incremental adaptation might no longer be suitable to respond to this kind of seismic, erratic changes. Instead, we might have to embrace a way of thinking that is itself radical and able to make big stretches and adjustments in very rapid sequence. We might no longer have the luxury of taking time to pause, notice, think and feel through things—perceptual, cognitive, and affective barriers might dissolve and no longer be relevant. There will likely be little or no time for resistance, objection or denial. Navigating such an unpredictable, high pace complexity will require the cultivation of an open mind, a flexible attitude, and readiness to act swiftly and collaboratively for the common good.

This attitude and skills are also needed to put things into perspective, devise new solutions and find resources and inspiration where we couldn’t previously see any. For instance, environmental geographer Lesley Head (2015, p. 314) invites us to look at our past as a resource: “the past also provides some imaginative resources to deal with what we currently think of as catastrophe, if we can free ourselves of teleological and progressivist framings of history.” As a case in point, the study of how societies adapted to past climate changes such as the Little Ice Age (the period of cold weather affecting Europe between the fourteenth and nineteenth centuries) might provide insights on how we can learn to cope with similar, if likely more radical transformations. Approaching the past through flexible, open-minded ‘ecological’ lenses opens up exciting new horizons and mandates for disciplines such as environmental history and the environmental humanities. In a similar vein, mental flexibility may be useful to ride the emotional turmoil of increasing ecological disasters. To this end, we might use rhetorical frameworks to recast negative emotions, whereby for instance “guilt and grief are framed as teachers, helping orient us toward love, care, and connection with the ecosystems that sustain life” (Jensen 2019, p. 123).

More broadly, mental flexibility might help us rethink our weakness in more positive terms. For instance, vulnerability has long been seen as synonymous of weakness, and contrasted to resilience. But what if we thought about vulnerability as a skill and a potential asset? As mentioned, awareness of vulnerability turns it into an asset. Specifically, awareness of one’s vulnerability can act as a threshold of alertness and foster more accurate appraisal. As Gibson et al. (2015, p. 418) put it “vulnerabilities inverted open up possibilities to identify creative abilities and capacities”. The authors suggest reframing difficulties and failures as ‘productive disruptions’(2015, p. 422), noting also that traumatic events often foster resourcefulness and expand social connections.

### Creativity

A consequence of mental openness and flexibility will likely be original, never-thought-before approaches and solutions. Creative minds are a gift, but they can also be nurtured, and we might wish to pay more attention to the education of children, taking more seriously their voices and responses to the world.

The last decades have shown the extent to which art and creativity are efficacious and much needed to make the loss visible and felt: whether it is through the visual arts, performance, films and documentaries, or the written word (as evidenced by the expanding fields of environmental narrative, ecocriticism, and ecopoetry). More specifically, we will need to be creative in devising ways to grieve nonhuman losses, to forge new social, cultural and political vocabularies to articulate these losses and make them grievable. We might have to conceive new visual and rhetoric languages, registers and practices that will carve out a space for the nonhuman in individual and collective awareness and affects. As Tim Jensen notes (2019, p. 128) “Expanding the boundaries of mournable bodies will require substantial rhetorical innovation in a variety of forms.” Grievability of nonhuman losses might also be achieved by fostering individuation, for instance through naming. Naming—giving a name to a plant, animal or landscape feature—fosters acknowledgment and through that, facilitates mourning. As Barnett (2019, p. 292) put it, “giving a name and uttering a name are ways of giving birth to a life which can be mourned.” As a condition for grievability in Judith Butler’s sense, naming “contains the seeds of care and concern which undergird compassionate, ethical relations” (Barnett 2019, p. 288). Importantly, naming also carries the potential to *engender* ecological awareness.

## The moral imperative of ecological grieving and mourning

The ecological crisis is at heart a crisis of emotions, as we fail to take notice, to emote, to express our feelings, share them with others, and build on this to act constructively. Not everyone mourns and grieves for ecological losses. Not everyone feels or expresses the pain in the same way. Nevertheless grieving and mourning are appropriate and necessary responses. Environmental philosopher Thom van Dooren (2014, p. 144) holds that “learning to mourn might offer us a way into a fuller understanding of our living planet, of what it means and why it matters”. Emotional responses—whether they are present or not, and their nature—are what make the difference between ecological awareness and a propensity for pro-environmental action, and indifference or denial. Public grieving and mourning for ecological destruction are effective ways to implement a change in direction. Despite the cold and the long hike, many people turned up for the memorial of the melted Okjokull Icelandic glacier in 2019. Prior to that, few of those people might have consciously paused to think about the relevance of the glacier in their lives, few might have actually hiked over it, and many might have just seen it from afar, or in a picture. Yet they showed up and mourned.

The paradox inherent in many kinds of losses is that we only become aware of the loss once it’s too late, when the loss makes itself manifest through the absence. The current ecological crisis impels us all now to move beyond this paradox: we must mourn all losses and we must mourn also for what is about to be lost, for what we don’t know is lost, for the ecosystems we have never known existed or never cared to learn about. If Aldo Leopold’s statement “we grieve only for what we know” (1949) might have been acceptable decades ago, it is no longer today. It is ethically imperative that we put an end to our collective indifference. As Braun (2017, p. 86) states “the incapacity to mourn is the indicator of not simply a loss of empathy for our environment, but the loss of kinship relations, of social meaning in general”. Experiencing and expressing grief for ecological loss has normative and ethical significance: it means that we value what surrounds us and the possibility to entertain a respectful and reciprocal relationship with it.

The consequences of ecological loss reach down to the ethical foundations of what makes us human, forcing us to question, with urgency, the lack of integrity that has brought us here. As the ecocide continues, collective inaction is tantamount to complicity. And so what we are losing in the process is also our innocence, our ability to say ‘I didn’t know’. We are mourning the loss of a certain notion of who we are, and who we have been, and beginning the process of re-constructing and re-defining our *Homo sapiens* identity. As Head (2015, p. 315) poignantly noted “If part of what we are grieving for, and what we must farewell, is our modern selves, it follows that a necessary intellectual and practical task is to imagine new kinds of selves.” This crucial task of redefining what it means to be a citizen of a post-Anthropocenic world must involve a new ethical covenant: abolishing all hierarchies among life forms.

Any vision of the future that aspires to be ethical needs to stem from looking square at the ecological loss we have caused: acknowledging it, paying respect to it, and feeling it—in short, grieving and mourning. These acts become then exemplary, they offer a model of behavior alternative to despair and indifference, and based instead on witnessing and taking responsibility. What might appear an obvious, matter-of-fact situation—earthly coexistence—is actually the result of labor, care, concern, engagement, dedication, perseverance. There is a sense in which ecological grief and mourning approximate a skill, an ability we can learn (Barnett 2022 talks of ‘achievement’); they point at other skills which will possibly define post-Anthropocenic scenarios—such as the ability to nourish intimacy, mental flexibility, and creativity. Ecological grief and mourning are crucial steps towards apprenticing ourselves to more ethical ways of relating, and towards giving new depth of meaning to present and future human and nonhuman life on the planet.

## Footnotes

1 For a comprehensive overview of climate-related emotions see Pihkala (2022).

2 Ecological grief is a complex emotion encompassing sorrow, anger, guilt and anxiety for the future among other emotions. For a sake of simplicity, I use the expression ‘ecological grief’ as an umbrella term referring to this broad range of emotional nuances.

3 https://www.lostspeciesday.org/.

4 https://www.floridamuseum.ufl.edu/science/rare-lichen-unique-to-florida-may-be-extinct/.

5 https://creatures-eu.org/mesosanctuary-mural-exploring-ecological-grief/.
